# When AI models take the exam: large language models vs medical students on multiple-choice course exams

**DOI:** 10.1080/10872981.2025.2592430

**Published:** 2025-11-29

**Authors:** Pablo Ros-Arlanzón, Renato Gutarra-Ávila, Vicente Arrarte-Esteban, Vicente Bertomeu-González, Luis Hernández-Blasco, Mar Masiá, Laura Navarro-Canto, Juan Nieto-Navarro, Javier Abarca, Angel P. Sempere

**Affiliations:** a Neurology Department, Dr. Balmis General University Hospital, Alicante, Spain; b Alicante Institute for Health and Biomedical Research (ISABIAL), Alicante, Spain; c Department of Clinical Medicine, Miguel Hernández University, Alicante, Spain; d Cardiology Department, Dr. Balmis General University Hospital, Alicante, Spain; e Cardiology Department, Hospital Clínica Benidorm, Benidorm, Spain; f Department of Pulmonology, Dr. Balmis General University Hospital, Alicante, Spain; g Infectious Diseases Unit, Hospital General Universitario de Elche, Spain; h Department of Neurology, Hospital General Universitario de Elche, Elche, Spain; i Department of Neurosurgery, Dr. Balmis General University Hospital, Alicante, Spain; j Department of Pathology and Surgery, Miguel Hernández University, Alicante, Spain

**Keywords:** Large language models, Artificial Intelligence, medical education, multiple-choice questions, medical students, ChatGPT, deepseek, copilot, gemini

## Abstract

Large language models (LLMs) are increasingly used in healthcare and medical education, but their performance on institution-authored multiple-choice questions (MCQs), particularly with negative marking, remains unclear. To compare the examination performance of five contemporary LLMs with enrolled medical students on final multiple-choice (MCQ-style) course exams across four clinical courses. We conducted a comparative cross-sectional study at Miguel Hernández University (Spain) in 2025. Final exams in Infectious Diseases, Neurology, Respiratory Medicine, and Cardiovascular Medicine were administered under routine conditions in Spanish. Five LLMs (OpenAI o1, GPT-4o, DeepSeek R1, Microsoft Copilot, and Google Gemini 1.5 Flash) completed all MCQs in two independent runs. Scores were averaged and test–retest was estimated with Gwet’s AC1. Student scores (*n* = 442) were summarized as mean ± SD or median (IQR). Pairwise differences between models were explored with McNemar’s test; student–LLM contrasts were descriptive. Across courses, LLMs consistently exceeded the student median and, in several instances, the highest student score. Mean LLM courses scores ranged 7.46–9.88, versus student means 4.28–7.32. OpenAI o1 achieved the highest mean in three courses; Copilot led in Cardiovascular Medicine (text-only subset due to image limitations). All LLMs answered every MCQ and short term test–retest agreement was high (AC1 0.79–1.00). Aggregated across courses, LLMs averaged 8.75 compared with 5.76 for students. On department-set Spanish MCQ exams with negative marking, LLMs outperformed enrolled medical students, answered every item, and showed high short-term reproducibility. These findings support cautious, faculty-supervised use of LLMs as adjuncts to MCQ assessment (e.g. automated pretesting, feedback). Confirmation across institutions, languages, and image-rich formats, and evaluation of educational impact beyond accuracy are needed.

## Introduction

Artificial intelligence (AI) is reshaping health care, from clinical decision support to diagnostic algorithms and personalized treatment [1]. AI also has the potential to transform medical education. A recent scoping review maps AI across the education continuum, from admissions to teaching and learning (intelligent tutoring systems, adaptive platforms, chatbots, and virtual patient simulators) and assessment [2].

Medical education depends on rigorous, standardized assessments to monitor knowledge growth and readiness for practice. Multiple-choice questions (MCQs) remain central because they enable broad sampling of curricular content, can be administered efficiently, and allow reliable computer-based scoring [3]. At the same time, AI chatbots have emerged as study aids, raising a practical question for educators: how well these systems perform on the same MCQ formats used in medical school curricula? [4].

Early evaluations showed that the initial version of ChatGPT could achieve near-passing scores on United States Medical Licensing Examination (USMLE) items [5]. Since then, performance on licensing-style MCQs has improved: a 2024 systematic review/meta-analysis reported that GPT−4 passed 26 of 29 medical exams and outperformed student averages in 13 of 17 comparisons, with results moderated by language, question difficulty, and prompt design (with higher performance on exams from English-speaking countries) [6]. A cross-cultural comparison of GPT−3.5, GPT−4, GPT-4o and Bard found GPT−4/GPT-4o generally superior to GPT−3.5 and Bard, with some context-dependent differences (e.g. image-based items, non-English exams) [7].

Head-to-head studies using undergraduate course MCQs are fewer but informative. At the University of the West Indies (Barbados), GPT−4 scored 73.7% vs 66.7% for Year 1 and Year 3 students [8]. In a medical biochemistry course (student year not specified), Claude, GPT−4, Gemini, and Copilot collectively averaged 81.1% and exceeded student performance by 8.3 percentage points (Claude > GPT−4 > Gemini > Copilot) [9].

Over the past year, reasoning-optimized models have emerged. The best-known of these are OpenAI o1 and DeepSeek R1, which have shown significant performance improvements on reasoning and planning tasks [10]. Recent benchmarking compared OpenAI o1, DeepSeek R1, and Llama 3.1-405B, across multiple medical tasks: on USMLE MCQs, OpenAI o1 achieved higher accuracy than DeepSeek R1(0.95 vs 0.92; *P* = 0.04), and both outperformed Llama 3.1-405B (0.83; *P* < 0.001) [11]. Gérard et al. evaluated GPT−4 Omni, Gemini Advanced, Le Chat, and DeepSeek R1 on institution-authored Clinical Pharmacology and Therapeutics examinations in France, reporting that DeepSeek R1 scored above third-year bachelor’s (L3) and first-year master’s (M1) students but below second-year master’s (M2) students [12]. Thus, while student-versus-model comparisons now include DeepSeek R1 in course settings, to our knowledge, no published study has directly compared enrolled students with OpenAI o1 and DeepSeek R1, tested side by side, on institution-authored, course-specific MCQ finals under routine conditions.

This gap provides the rationale for our study. We conducted a comparative, head-to-head evaluation at Miguel Hernández University, comparing GPT-4o, OpenAI o1, DeepSeek R1, Google Gemini 1.5 Flash, and Microsoft Copilot with enrolled medical students. The evaluation used final examinations composed exclusively of single-best-answer MCQs across multiple clinical courses, all administered under routine conditions and scored with negative marking.

## Material and methods

### Study design

We conducted a comparative, cross-sectional study between January and February 2025 at Miguel Hernández University (Spain). The objective was to evaluate the examination performance of five LLMs: OpenAI o1, GPT-4o, DeepSeek R1, Microsoft Copilot, and Google Gemini 1.5 Flash, on final MCQ examinations from four medical-school courses commonly named in U.S. curricula as Infectious Diseases, Neurology, Respiratory Medicine, and Cardiovascular Medicine, and to compare model performance with that of enrolled third and fourth-year medical students. All items had four options and a single correct answer, administered in Spanish. All exams formed part of the required summative assessment for each course and were mandatory for enrolled students. Prior similar exams had been validated in prior years by course faculty, showing acceptable item discrimination and difficulty indices, consistent with institutional standards.

Exam results were expressed according to the Spanish university grading scale (0.00−10.00).Incorrect answers incurred penalties according to a course-specific correction factor R. R = 0.33 for Neurology and Cardiovascular Medicine, R = 0.25 for Infectious Diseases and Respiratory Medicine. Unanswered items were not penalized. Scores were computed as: Score = [Correct − (Incorrect × R)] × (10/Total items).

### LLM evaluation

Exam items were accessed by one of the study authors (RGA), who manually input each question to the LLMs through their respective native interfaces under default settings. To minimize context carryover, a new session was started for each item whenever the platform allowed (all models except Gemini, which did not support session resets). Two independent runs were conducted to estimate short-term reproducibility, given that model responses may vary due to inherent stochasticity. No explicit time limit was imposed on LLMs. A standardized prompt was used:

"Please complete a test on [course]. The questions are in Spanish, each with four options and only one correct answer. Incorrect answers are penalized by [0.25 or 0.33]. If unsure, omit answer. Try to maximize your final score."

Prompt optimization was not explored, as the goal was to evaluate each model’s default reasoning capability under standardized, reproducible conditions. Using default settings enhances comparability and reflects typical user behavior in educational contexts [13].

Due to platform limitations, image-based questions were excluded for Gemini and Copilot in the Cardiovascular exam. To assess short-term stability, each exam was re-administered once to each model 1–7 days after the first attempt, using the same items, order, and prompt.

### Data analysis

Student score distributions were assessed for normality with the Kolmogorov–Smirnov test (*α* = 0.05). Student performance was summarized as mean scores with 95% confidence intervals (CIs). For each LLM and course, the primary descriptive estimate was the mean of two attempts. Inter-test agreement for each model and course was quantified with Gwet’s AC1 [14]. We compared the performance of LLMs on MCQs using McNemar’s test. For each specialty, the responses of each model were transformed into a binary variable indicating whether the answer was correct or incorrect. McNemar’s test was then applied to paired data to evaluate whether there was a significant difference in the proportion of correct answers between the two models. Statistical analyzes were conducted using R (v4.5.1) and RStudio (v2025.05.1 +513). Given the observational design and characteristics of the study, comparisons between students and LLMs were descriptive rather than inferential. Although we refrained from formal inferential testing between students and LLMs due to design and distributional considerations, future studies could strengthen interpretation by applying regression or effect-size analyzes to account for course difficulty or other potential confounders. Statistical analyzes were performed by two study authors (PRA and APS - faculty with experience in medical statistics).

### Ethical considerations

The study protocol was approved by the Ethics and Research Integrity Committee, Miguel Hernández University (Approval Code: TFG.GME.ACPS.RPGA.241221). Student records were anonymized and analyzed as secondary data; individual informed consent was not required.

## Results

We analyzed 442 exam scores from third- (*n* = 114) and fourth-year (*n* = 328) medical students across four clinical courses, Infectious Diseases, Neurology, Respiratory Medicine, and Cardiovascular Medicine, and contrasted them descriptively with five LLMs (OpenAI o1, GPT-4o, DeepSeek R1, Microsoft Copilot, and Google Gemini). Each LLM answered all MCQs; two independent runs were performed per model. Across courses, LLM scores typically exceeded the student median and, in several instances, the highest student score.

### Respiratory medicine

**Figure 1.**
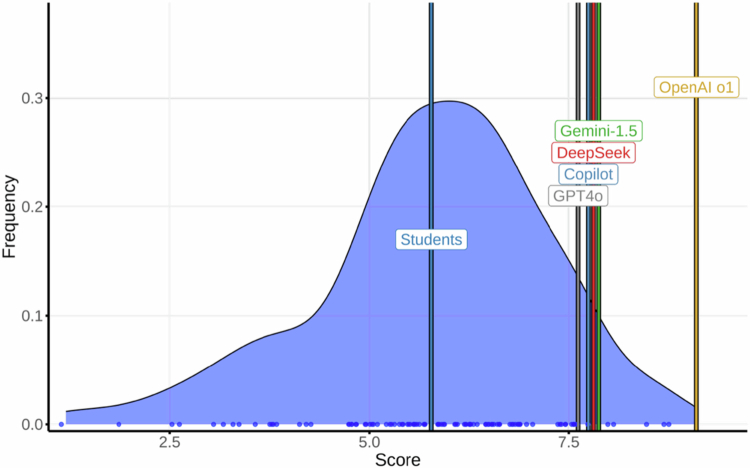
Distribution of final exam scores in Respiratory Medicine among medical students and Large Language Models (LLMs).

Students achieved a mean score of 5.78 (95% CI, 2.55–8.31; max 8.70). LLMs scored as follows: OpenAI o1, 9.04 (both attempts); Gemini, 7.73; DeepSeek, 7.66; Copilot, 7.60; and GPT-4o, 7.46 (LLM mean: 7.90). These results are illustrated in Figure 1. Test–retest agreement (AC1) for the two OpenAI o1 runs was 0.96; for other models: GPT-4o, 0.95; Copilot, 0.93; Gemini, 0.88; and DeepSeek, 0.79. Only OpenAI o1 and Copilot maintained or improved their score on retest.

### Neurology

**Figure 2.**
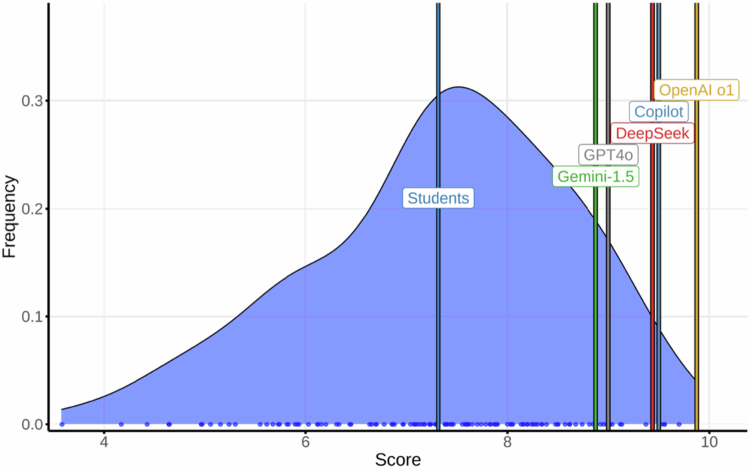
Distribution of final exam scores in Neurology among medical students and Large Language Models (LLMs).

Students achieved their highest scores in this course (mean 7.32; 95% CI, 4.56–9.47; max 9.73). The LLMs consistently outperformed students: OpenaI o1, 9.87; Copilot, 9.47; DeepSeek, 9.40; GPT-4o, 8.94; and Gemini, 8.80 (Figure 2) (LLM mean: 9.30). Test–retest agreement (AC1): OpenAI o1 and Copilot, 1.00; GPT-4o and DeepSeek, 0.92; Gemini, 0.89. All models except DeepSeek and Gemini maintained or improved their score on retest.

### Infectious diseases

**Figure 3.**
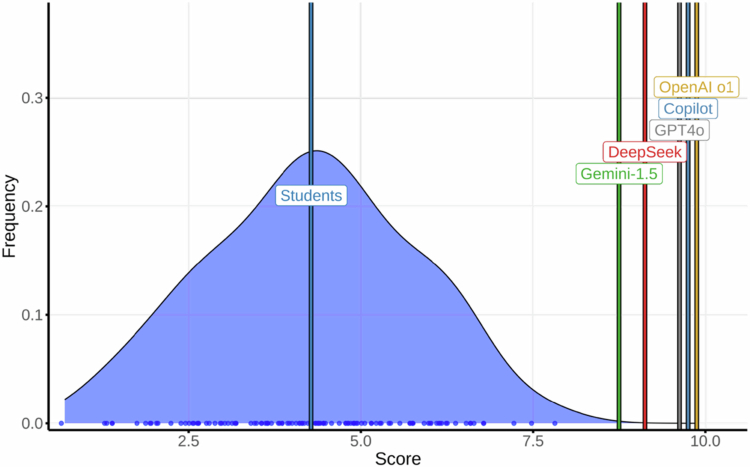
Distribution of final exam scores in Infectious Diseases among medical students and Large Language Models (LLMs).

This was the lowest-performing course for students (mean 4.28; 95% CI, 1.39–6.79; max 7.85). The LLMs scored highly: OpenAI o1, 9.88 (10.0 on retest); Copilot, 9.75; GPT-4o, 9.63; DeepSeek, 9.13; and Gemini, 8.75 (Figure 3) (LLM mean: 9.43). Test–retest agreement (AC1): Copilot, 1.00; OpenAI o1 and GPT-4o, 0.98; and DeepSeek and Gemini, 0.92. All models maintained or improved their performance on the second attempt.

### Cardiovascular medicine

**Figure 4.**
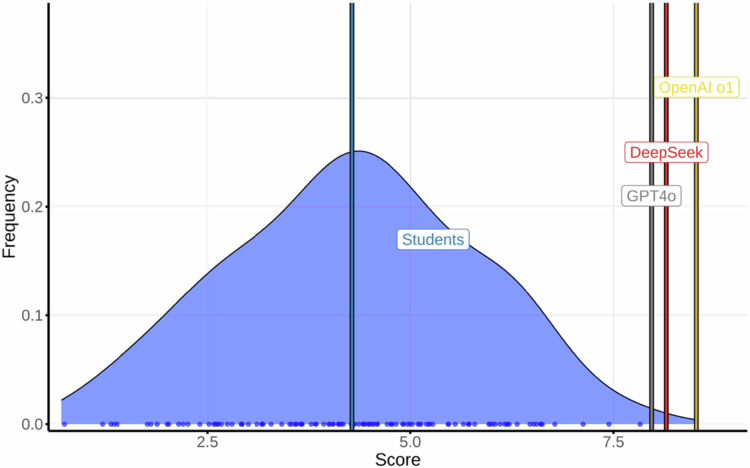
Distribution of final exam scores in Cardiovascular Medicine among medical students and Large Language Models (LLMs).

Students averaged 5.28 (95% CI, 2.22–7.90; max 8.15). The LLMs scores were: Copilot, 9.11; Gemini, 8.67; OpenAI o1, 8.61; DeepSeek, 8.26; and GPT-4o, 8.09 (Figure 4) (LLM mean: 8.55). Test–retest agreement (AC1): OpenAI o1, 1.00; GPT-4o, 0.97; DeepSeek, 0.94; Gemini, 0.93; and Copilot, 0.88. As noted in Methods, image-based items in this course could not be rendered for Gemini and Copilot, so their scores reflect a text-only subset. All models except DeepSeek maintained or improved their score on retest.

### Overall performance

**Figure 5.**
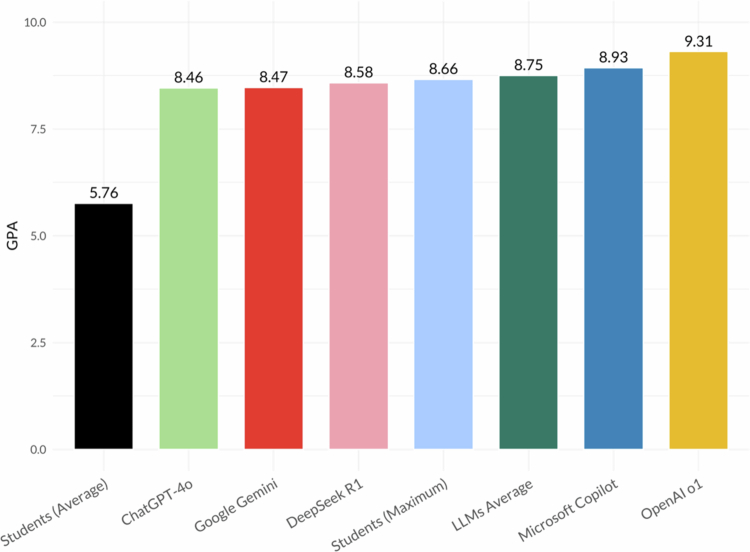
Overall performance of large language models (LLMs) compared with medical students. Bar chart of mean exam scores (0−10) for each LLM, the student ´ s mean and maximum scores, and the mean across LLMs, ordered from lowest to highest.

Aggregated across courses, OpenAI o1 achieved the highest mean score (9.31), followed by Copilot (8.93), DeepSeek (8.58), Gemini (8.47), and GPT-4o (8.46). The student cohort’s weighted average was 5.76 (max 8.66), and the mean across all LLMs was 8.75. These results are presented descriptively to illustrate overall performance differences (Figure 5). Global test–retest agreement (AC1) was highest for OpenAI o1 (0.985), followed by GPT-4o (0.955), Copilot (0.951), Gemini (0.904), and DeepSeek (0.895). Detailed performance for each course, including student means and maxima, as well as LLM AC1 values, are provided in Supplementary Table 1.

**Figure 6.**
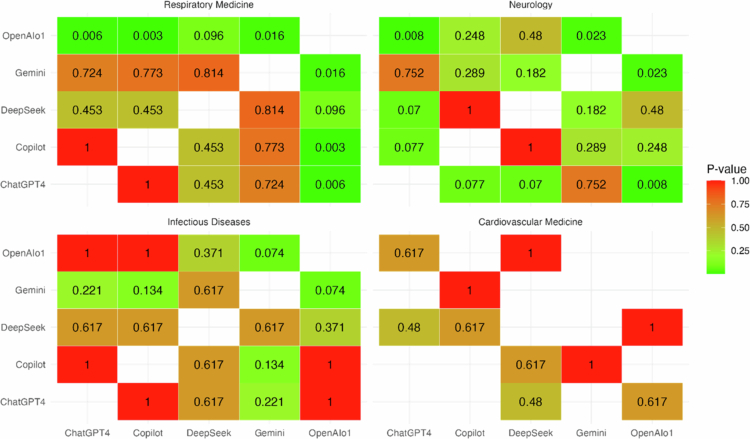
Heatmap of pairwise McNemar p-values for model responses across medical specialties. Each tile represents the p-value for the comparison between two models; lower p-values (green) indicate significant differences, higher p-values (red) indicate no significant difference.

McNemar’s test was applied to the binary outcomes (correct vs. incorrect) for each pair of models (Figure 6). In Respiratory Medicine, OpenAI o1 performed significantly better than ChatGPT−4 (*p* = 0.006), Gemini (*p* = 0.016), and Copilot (*p* = 0.003), while the comparison with DeepSeek showed only a nonsignificant trend (*p* = 0.096); no other pairwise differences were significant. In Neurology, OpenAI o1 outperformed ChatGPT−4 (*p* = 0.008) and Gemini (*p* = 0.023), with all other comparisons, including Copilot vs. OpenAI o1 (*p* = 0.248) and DeepSeek vs. OpenAI o1 (*p* = 0.480), showing no statistical differences. In Infectious Diseases, OpenAI o1 showed a nonsignificant trend toward better performance compared with Gemini (*p* = 0.074), but no pairwise comparisons reached significance. In Cardiovascular Medicine, because of limited comparability (Gemini and Copilot were text-only), fewer comparisons were possible, and no significant differences were observed. For the complete data, including the number of discordant responses and the corresponding *p*-values, see Supplementary Table 2.

## Discussion

Using medical-school final examinations administered under routine conditions, in Spanish, and scored with negative marking, we found that five LLMs outperformed the student cohort in Infectious Diseases, Neurology, Respiratory Medicine, and Cardiovascular Medicine. LLMs surpassed the students’ mean score and, in some instances, the highest student score. OpenAI o1 achieved the highest mean in three of four courses (Infectious Diseases, Neurology, and Respiratory Medicine), whereas Microsoft Copilot led in Cardiovascular Medicine (limited to a text-only subset due to platform image limitations). Notably, all five models answered every MCQ, and short-term test–retest agreement was high for all (highest AC1 for OpenAI o1), indicating reproducible performance that was not driven by selective abstention.

These results extend prior work from licensure-style evaluations, which demonstrated rapid improvement from early ChatGPT to ChatGPT−4, as well as pass-level performance into locally authored medical-school exams with penalties for incorrect answers [5–7]. Course-level studies similarly report that LLMs surpass students in undergraduate settings [9,15]. Similar results have been explored in postgraduate and discipline-specific evaluations, including radiology and neurology examinations, where recent studies reported variable but increasingly competitive LLM performance [16–18].

New reasoning-optimized systems, such as OpenAI o1 and DeepSeek R1, have outperformed previous LLMs. In a U.S. study using ophthalmology board-style questions, OpenAI o1 was superior to GPT-4o, Gemini, and human test-takers [19]. DeepSeek-R1, a free and open-source LLM from China, demonstrated superior performance compared with GPT-4o on the Chinese National Medical Licensing Examination [20], and performed similarly to GPT-4o in the Polish Infectious Diseases specialty exam [21].

Head-to-head comparisons of OpenAI o1 and DeepSeek R1 have shown mixed results. In two English-language evaluations (USMLE MCQs and U.S. ophthalmology board-style MCQs), OpenAI o1 outperformed DeepSeek R1 [11,22]. In a bilingual Chinese/English ophthalmology exam derived from the Chinese professional title test, DeepSeek R1 surpassed OpenAI o1 [23]. In a 300-case ophthalmology-focused study, DeepSeek R1 showed strong diagnostic and management performance comparable to OpenAI o1, at lower cost, though access differed (DeepSeek R1 via public chat and OpenAI o1 via Application Programming Interface) [24]. Our findings extend this evidence to Spanish-language, course-final MCQs with negative marking, where OpenAI o1 again ranked above DeepSeek R1.

Importantly, our exams were authored by course faculty rather than drawn from public question banks. This means that distractors were tailored to the local cohort, making the observed LLM advantage more relevant to the authentic evaluative practices of medical schools. Such performance goes beyond standardized benchmarks, suggesting that current LLMs are competitive not only with external test sets but also with in-house examinations designed to challenge students at their own institution. Taken together, the literature and our findings suggest that reasoning-optimized LLMs, particularly OpenAI o1 now perform robustly under assessment conditions relevant to medical schools.

Medical education doesn’t have the luxury of watchful waiting; the field needs to grapple now with the effects of AI [25]. Given their high accuracy under negative marking and universal answering, LLMs might be used as faculty-supervised adjuncts in MCQ assessment. Automated pretesting can rapidly identify flawed items, misleading stems, overlapping options, weak or non-functioning distractors, and questions solvable via superficial cues, thereby improving validity before administration. Beyond item quality, supervised use of LLMs could also provide formative feedback to learners and support curriculum development.

Strengths of this study include the use of medical-school final examinations, negative marking across courses, head-to-head comparison between students and LLMs, inclusion of five contemporary models (notably OpenAI o1 and DeepSeek R1), and two attempts per model with AC1 stability estimates. Limitations include single-institution scope and Spanish-language items, which may limit generalizability to other educational contexts, languages, or exam format -particularly those with image-rich questions. The exclusion of image-based items for Gemini and Copilot in Cardiovascular Medicine further constrains cross-model comparability.

Our study relied on traditional MCQs, which remain central to medical-school assessment. However, MCQs may not fully capture LLM reasoning ability compared with free-response or multi-turn formats. Future work should include multi-institution replication across languages, paired text-only vs image-rich questions to isolate modality effects, and extend benchmarking to newer LLMs (e.g. ChatGPT−5, Gemini 2.5, DeepSeek-R1−0528). Beyond accuracy, it will also be important to assess the educational quality of model explanations and their impact on learner outcomes.

## Conclusions

In department-set, Spanish medical-school final MCQ exams with uniform negative marking, five LLMs, notably OpenAI o1, outperformed enrolled students, answered every item, and showed high short-term reproducibility. These findings support guarded, faculty-supervised use of LLMs as adjuncts to MCQ assessment (e.g. automated pretesting and feedback). Confirmation across institutions, languages, and image-rich formats is needed before broader adoption.

## Supplementary Material

Supplementary Material Supplementary Tables

## Data Availability

The data that support the findings of this study are available from the corresponding author upon reasonable request.
