# Supplementary material for: When AI models take the exam: large language models vs medical students on multiple-choice course exams
Source: Med Educ Online. 2025 Nov 29;30(1):2592430. doi: 10.1080/10872981.2025.2592430 (PMC12667333; doi:10.1080/10872981.2025.2592430)
Supplement: Supplementary Material — Supplementary Tables [file ZMEO_A_2592430_SM1606.docx]

**APPENDIX**

**Supplementary Table 1. Performance of Large Language Models and Medical Students on Departmental Final MCQ Exams Across Clinical Courses**

| **LLM / Students** | **Pulmonary Medicine** | **Neurology** | **Infectious Diseases** | **Cardiovascular Medicine** | **Global Test–Retest Agreement (AC1)** |
| --- | --- | --- | --- | --- | --- |
| Students (Mean, 95% CI) | 5.78 (2.55–8.31) | 7.32 (4.56–9.47) | 4.28 (1.39–6.79) | 5.28 (2.22–7.90) | N/A |
| Students (Maximum) | 8.70 | 9.73 | 7.85 | 8.15 | N/A |
| OpenAI o1 | 9.04 | 9.87 | 9.88 | 8.61 | 0.985 |
| DeepSeek R1 | 7.66 | 9.40 | 9.13 | 8.26 | 0.895 |
| ChatGPT-4o | 7.46 | 8.94 | 9.63 | 8.09 | 0.955 |
| Microsoft Copilot | 7.60 | 9.47 | 9.75 | 9.11 | 0.976 |
| Google Gemini | 7.73 | 8.80 | 8.75 | 8.67 | 0.895 |

Scores reflect final MCQ exam performance (maximum 10 points) in each clinical course. Global Test–Retest Agreement (AC1) was calculated for each LLM across two independent attempts. CI = confidence interval; N/A = not applicable.

Supplementary Table 2. McNemar test results for pairwise comparisons of model responses across specialties.

| **Specialty** | **Comparison** | **Discordant Responses (0→1 / 1→0)** | **P-value** |
| --- | --- | --- | --- |
| Respiratory Medicine | ChatGPT4 vs Gemini | 5 / 3 | 0.724 |
|  | ChatGPT4 vs Copilot | 5 / 5 | 1.000 |
|  | ChatGPT4 vs DeepSeek | 10 / 6 | 0.453 |
|  | ChatGPT4 vs OpenAIo1 | 12 / 1 | 0.006 |
|  | Gemini vs Copilot | 5 / 7 | 0.773 |
|  | Gemini vs DeepSeek | 10 / 8 | 0.814 |
|  | Gemini vs OpenAIo1 | 10 / 1 | 0.016 |
|  | Copilot vs DeepSeek | 10 / 6 | 0.453 |
|  | Copilot vs OpenAIo1 | 11 / 0 | 0.003 |
|  | DeepSeek vs OpenAIo1 | 10 / 3 | 0.096 |
| Neurology | ChatGPT4 vs Gemini | 6 / 4 | 0.752 |
|  | ChatGPT4 vs Copilot | 7 / 1 | 0.077 |
|  | ChatGPT4 vs DeepSeek | 9 / 2 | 0.070 |
|  | ChatGPT4 vs OpenAIo1 | 9 / 0 | 0.008 |
|  | Gemini vs Copilot | 6 / 2 | 0.289 |
|  | Gemini vs DeepSeek | 7 / 2 | 0.182 |
|  | Gemini vs OpenAIo1 | 7 / 0 | 0.023 |
|  | Copilot vs DeepSeek | 3 / 2 | 1.000 |
|  | Copilot vs OpenAIo1 | 3 / 0 | 0.248 |
|  | DeepSeek vs OpenAIo1 | 2 / 0 | 0.480 |
| Infectious Diseases | ChatGPT4 vs Gemini | 1 / 5 | 0.221 |
|  | ChatGPT4 vs Copilot | 1 / 1 | 1.000 |
|  | ChatGPT4 vs DeepSeek | 1 / 3 | 0.617 |
|  | ChatGPT4 vs OpenAIo1 | 2 / 1 | 1.000 |
|  | Gemini vs Copilot | 4 / 0 | 0.134 |
|  | Gemini vs DeepSeek | 3 / 1 | 0.617 |
|  | Gemini vs OpenAIo1 | 5 / 0 | 0.074 |
|  | Copilot vs DeepSeek | 1 / 3 | 0.617 |
|  | Copilot vs OpenAIo1 | 1 / 0 | 1.000 |
|  | DeepSeek vs OpenAIo1 | 4 / 1 | 0.371 |
| Cardiovascular Medicine | ChatGPT4 vs DeepSeek | 2 / 0 | 0.480 |
|  | ChatGPT4 vs OpenAIo1 | 3 / 1 | 0.617 |
|  | DeepSeek vs OpenAIo1 | 2 / 2 | 1.000 |
|  | Gemini vs Copilot | 4 / 4 | 1.000 |

Discordant responses are presented as “0→1 / 1→0”, where 0 denotes an incorrect answer and 1 a correct answer. Thus, “0→1” indicates the number of cases where Model 1 was incorrect and Model 2 correct, and “1→0” the opposite situation. P-values correspond to McNemar’s test for each comparison.
